# Influence of fat on liver *T*_1_ measurements using modified Look–Locker inversion recovery (MOLLI) methods at 3T

**DOI:** 10.1002/jmri.25146

**Published:** 2016-01-13

**Authors:** Ferenc E. Mozes, Elizabeth M. Tunnicliffe, Michael Pavlides, Matthew D. Robson

**Affiliations:** ^1^ University of Oxford Centre for Clinical Magnetic Resonance Research (OCMR)University of Oxford, John Radcliffe HospitalOxfordUK; ^2^ Translational Gastroenterology UnitUniversity of Oxford, John Radcliffe HospitalOxfordUK

**Keywords:** MOLLI T1 map, bSSFP, fatty liver disease

## Abstract

**Purpose:**

To characterize the effect of fat on modified Look–Locker inversion recovery (MOLLI) *T*_1_ maps of the liver. The balanced steady‐state free precession (bSSFP) sequence causes water and fat signals to have opposite phase when repetition time (TR) = 2.3 msec at 3T. In voxels that contain both fat and water, the MOLLI *T*_1_ measurement is influenced by the choice of TR.

**Materials and Methods:**

MOLLI *T*_1_ measurements of the liver were simulated using the Bloch equations while varying the hepatic lipid content (HLC). Phantom scans were performed on margarine phantoms, using both MOLLI and spin echo inversion recovery sequences. MOLLI *T*_1_ at 3T and HLC were determined in patients (*n* = 8) before and after bariatric surgery.

**Results:**

At 3T, with HLC in the 0–35% range, higher fat fraction values lead to longer MOLLI *T*_1_ values when TR = 2.3 msec. Patients were found to have higher MOLLI *T*_1_ at elevated HLC (*T*_1_ = 929 ± 97 msec) than at low HLC (*T*_1_ = 870 ± 44 msec).

**Conclusion:**

At 3T, MOLLI *T*_1_ values are affected by HLC, substantially changing MOLLI *T*_1_ in a clinically relevant range of fat content. J. Magn. Reson. Imaging 2016;44:105–111.

THE MODIFIED LOOK–LOCKER INVERSION RECOVERY (MOLLI)^1^ *T*_1_ mapping technique and variants of it have been gaining interest in cardiovascular magnetic resonance imaging (MRI)^2^ and in liver imaging.^3^ MOLLI is able to build a *T*_1_ map within a breath‐hold. It has also been demonstrated that MOLLI *T*_1_ maps provide diagnostic information in the heart^4^ and correlate with liver fibrosis and inflammation.^3^

Fat is known to have a short *T*_1_, and in regions of visceral fat the MOLLI *T*_1_ method measures this short *T*_1_ with high reproducibility.^5^ Similarly, the MOLLI *T*_1_ method detects long *T*_1_ regions of the blood pool with high reproducibility.^6^ Patients suffering from liver‐related diseases have been shown to have fat values in the 2% to 44% range^7^, ^8^, ^9^; thus, in livers with large amounts of fat, simplified reasoning would predict that the measured *T*_1_ would be the weighted sum of the *T*_1_ of the fat and the *T*_1_ of the hepatic tissue. The measured *T*_1_ of the liver would thus be expected to be reduced in fatty liver due to this partial volume effect. Phantom scans and previous work have demonstrated that *T*_1_ decreases with increasing fat concentration when using conventional imaging methods, ie, spin echo inversion recovery (SE‐IR).^10^

MOLLI *T*_1_ values have been shown to be influenced by fat and off‐resonance frequencies at 1.5T in the calf muscle^11^ and the myocardium^12^ and elevated MOLLI *T*_1_ is often measured in patients with fatty livers^3^ at 3T, where balanced steady‐state free precession (bSSFP) repetition times (TRs) between 2.1 msec and 2.6 msec are commonly used.^3^, ^13^ It is the *T*_1_ of the water component which appears to be of diagnostic significance when using *T*_1_ mapping in the liver,^14^ while the *T*_1_ of the fat is constant^15^ and not a predictor of disease. An important step towards the use of MOLLI *T*_1_ to assess liver disease is the clarification and quantification of the extent to which the presence of fat can mask or exaggerate changes in MOLLI *T*_1_ due to disease.

The aim of this study was thus to investigate the effect of different fat concentrations and off‐resonance frequencies on MOLLI *T*_1_ maps at 3T with a bSSFP TR = 2.3 msec and to explain the mechanisms for this behavior.

## Theory

MOLLI is an electrocardiogram (ECG)‐gated inversion recovery *T*_1_ mapping method. It uses several inversion pulses and acquires images synchronized to the cardiac cycle using a snapshot bSSFP^16^ readout. Readouts are followed by pauses of a few heartbeats, allowing for full recovery of the longitudinal magnetization.^1^

**Figure 1:**
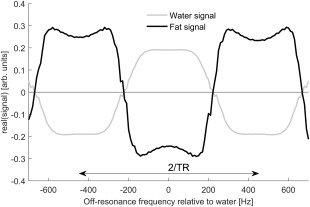
Variation of the steady‐state signal over off‐resonance frequencies relative to the water, as seen in Refs. 17 and 34. When a TR equal to the inverse of the chemical shift difference between water and fat is used, the steady‐state signal of fat and water will have opposite phases.

The bSSFP readout results in images that are sensitive to the off‐resonance frequency of the protons being imaged (Fig. 1).^17^ The chemical shift difference between water and the main fat peak is 3.5 ppm,^18^ which at 3T translates into a frequency difference of ∼447 Hz. Therefore, if bSSFP images are acquired with a repetition time close to 1/447 seconds (∼2.23 msec), the fat and water signals will have exactly opposite phase. When a different TR is chosen, the phase relationship between water and fat is a more complex function of off‐resonance frequency.

**Figure 2:**
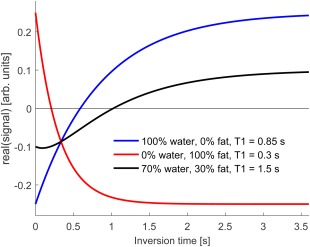
Schematic MOLLI recovery curves of water and fat components at identical main static field strength using TR = 2.3 msec. The 100% fat curve appears inverted because of its frequency shift. The different *T*_1_ recovery times of fat and water and their opposing phases at TR = 2.3 msec lead to an apparent lengthening of the *T*_1_ recovery in the combined signal.

The signal in a voxel containing both fat and water with no exchange of magnetization between them would be determined by the well‐known partial volume effect. If the fat and water were in phase then the recovery would follow the sum of these two recovering exponentials; in practice, if this were fit to a single exponential then the measured *T*_1_ would lie somewhere between the individual *T*_1_s of the fat and the water. However, in the situation described in this work the water and fat are exactly out of phase, and so the signal recovery is the difference of two exponentials weighted by the relative contributions of fat and water. In practice, for relatively small fat concentrations this results in a measured value for *T*_1_ that is longer than the *T*_1_ value of either the fat or water component.^19^ A conceptual schematic of this behavior is illustrated in Fig. 2.

## Materials and Methods

### Simulations

A Bloch equation^20^ simulation was built in MatLab (MathWorks, Natick, MA) which emulated the exact pulse sequence of a MOLLI acquisition on our scanner. The simulated sequence included the waveforms of radiofrequency (RF) inversion, preparation, and imaging pulses. The measured signal intensity for each simulation was determined as the signal averaged over a set of phase encode lines centered on k = 25. Magnetization transfer effects were not included in the simulation, although they are known to bias *T*_1_ with the MOLLI method.^21^

A single inversion followed by five image readouts was simulated, providing a good approximation to the MOLLI acquisition but eliminating the complexity that is due to the multitude of possible sampling schemes that have been described.^21^ Since liver MOLLI *T*_1_ values lie in the 700–1400 msec interval,^3^ five samples over the recovery curve were used for determining the *T*_1_.

The following simulation parameters were used: 3T field strength, 192 × 144 matrix, 84 phase encode lines, 24 phase encode lines before the central line, flip angle (FA) = 35°, TR/TE = 2.3/1.15 msec, TI = 100, 1000, 1900, 2800, 3700 msec, adiabatic hyperbolic secant 1 inversion pulse of 10.24 msec duration, time‐bandwidth product of R = 5.48, peak B_1_ = 750 Hz and β = 3.45.^21^ The bSSFP readout used five linearly increasing startup angle (LISA) pulses^22^, ^23^ and one half‐angle ramp down pulse.

*T*_1_ and *T*_2_ values for fat and liver components were: *T*_1fat_ = 382 msec, *T*_2fat_ = 68 msec,^24^ *T*_1hepatic_ = 812 msec,^25^ *T*_2hepatic_ = 34 msec.^24^ Separate signals corresponding to each component of a multiple‐peak lipid spectrum were weighted by their relative amplitude provided by Hamilton et al^26^ and summed to obtain a single fat signal. Simulated fat and water signals were combined in different proportions according to the principle of partial volumes and to reflect HLCs in the 0–100% range in steps of 1%, fat fractions, *F*, being defined using Eq. (1), where ρ_f_ and ρ_w_ denote the proton densities of fat and water.

(1) $$F=\frac{\rho_{f}}{\rho_{f}+\rho_{w}}\times 100[\%]$$

Then, the signal points were fitted to an exponential describing longitudinal recovery, given by Eq. (2), where *T*_1_* is the apparent *T*_1_ and A and B are additional fit parameters.

(2) $$S(TI)=A-B\text{exp}\left(-\frac{TI}{T_{1}^{*}}\right)$$

The MOLLI *T*_1_ values were computed by using the imperfect,^27^ but commonly used, Look–Locker correction method described by Eq. (3).^1^

(3) $$T1=T_{1}^{*}\left(\frac{B}{A}-1\right)$$

In order to show the changes in MOLLI *T*_1_ caused by repetition times other than 2.3 msec, the variation of MOLLI *T*_1_ with off‐resonance frequency was simulated for two repetition times found in the literature: TR = 2.14 msec^3^ and TR = 2.6 msec.^13^ All simulation parameters were the same as described above for the liver simulation, with the simplification that only three lipid concentrations were simulated: 0%, 10%, and 20%. The chosen off‐resonance frequency range was –100 Hz to 100 Hz in steps of 2 Hz, which covers the typical frequency range encountered in the in vivo MOLLI data discussed below.

To further explore the effects of fat on MOLLI *T*_1_ values, the TR was varied in a simulation comprising a water and a multiple‐peak fat component with 30% fat fraction. The spectral model of the fat was based on the ^1^H spectrum of the margarine phantom described below. The same imaging protocol was simulated as described in the previous section, with the following differences: TI = 200, 1200, 2200, 3200, 4200 msec, RR = 1000 msec, and 31 bSSFP TR values in the range 1.93 to 5.75 msec. *T*_1_ and *T*_2_ values for fat and water components corresponded to those of a margarine phantom and were *T*_1fat_ = 325 msec, *T*_2fat_ = 120 msec, *T*_1water_ = 2448 msec, and *T*_2water_ = 207 msec. The measurements leading to these values are described in the next section.

### Phantom Scans

Phantom experiments were carried out using a margarine phantom (Flora Light, Unilever) with 30% fat content. First, the margarine phantom was scanned using a 5‐point MOLLI sequence. Imaging parameters followed a standardized MOLLI acquisition protocol (Siemens WIP 561a, Erlangen, Germany): 192 × 144 matrix, FA = 35°, TR/TE = 2.3/0.99 msec, TI = 100, 1100, 2100, 3100, 4100 msec, simulated ECG with RR = 1000 msec. Then the same margarine phantom was scanned using the SE‐IR sequence. Imaging parameters followed a standardized acquisition protocol: 128 × 128 matrix, TR = 10000 msec, TI = 50, 150, 250, 400, 600, 900, 1300, 2000, 3000, 4500, 6500 msec, TE = 7.4 msec. In both cases images were acquired using a Siemens 3T Verio scanner using a 32‐channel head RF coil.

Following the margarine scans, a sample of the margarine was heated and then spun with a centrifuge (Rotanta 460R, Hettich) at 4000 rpm for 5 minutes. After spinning the sample, separate layers of fat and water were obtained that were scanned using the MOLLI acquisition and the SE‐IR acquisition using the same scanner and same protocols as described above.

A subsequent experiment was carried out in order to determine MOLLI *T*_1_ of the whole margarine and the water component only over a range of repetition times. The previously described 5‐point MOLLI acquisition protocol was used with the following changes: TI = 200, 1200, 2200, 3200, 4200 msec, RR = 1000 msec, and 31 bSSFP TR values ranging from 1.93 to 5.75 msec.

MOLLI *T*_1_ values were determined by fitting the signal intensity of a circular region of interest on the bSSFP images following the inversion pulse to Eq. (2), then applying Eq. (3) to obtain *T*_1_. *T*_1_ values for images acquired using the SE‐IR sequence were computed by fitting Eq. (4) to mean signal values sampled from the same region of interest in each image.

(4) $$S(TI)=C\left(1-2\text{exp}\left(-\frac{TI}{T_{1}}\right)+\text{exp}\left(-\frac{TR}{T_{1}}\right)\right)$$

### Patient Studies

In order to compare the results of the simulations to results obtained in vivo, data from eight patients (seven female, mean age: 49 ± 10.5 years) were processed retrospectively. Patients were scanned before weight reduction surgery and 6 months postoperatively. Relevant parameters of the patients before surgery included mean body mass index (BMI): 45.8 ± 5.5 kg/m^2^, mean waist circumference: 120.25 ± 10.35 cm, and mean hip circumference: 134.5 ± 11.2 cm. Four of the patients suffered from diabetes. Postoperative parameters were: mean BMI: 36.4 ± 4.8 kg/m^2^, mean waist circumference: 107.7 ± 10.6 cm, and mean hip circumference: 120.6 ± 8.6 cm.

The study protocol conformed to the ethical guidelines of the 1975 Declaration of Helsinki, and was approved by the institutional research departments and the National Research Ethics Service (13/SC/0243). All patients gave written informed consent.

At each timepoint, patients had liver MOLLI *T*_1_ maps (5‐point MOLLI was employed). *T*_2_* maps were collected for hepatic iron quantification and ^1^H MRS for the quantification of liver fat using the stimulated echo acquisition mode (STEAM) sequence. Imaging parameters for the MOLLI scan were: 192 × 134–160 matrix (depending on patient), field of view (FOV) 280–348 × 350–500 mm (depending on patient), slice thickness of 8 mm, GRAPPA acceleration factor 2, pixel bandwidth 898 Hz/px, TR/TE = 2.14/1.07 msec, FA = 35°, TI = 110, 110+RR, 110+2RR, 110+3RR, 110+4RR msec. MOLLI *T*_1_ maps were acquired in transverse scan planes.

*T*_2_* maps were determined using a multiecho acquisition with RF spoiling. Imaging parameters for this acquisition were as follows: same FOV as for the 5‐point MOLLI sequence, 192 × 128–160 matrix (depending on patient), slice thickness of 3 mm, GRAPPA acceleration factor 2, TR/TE = 26.5/2.46, 7.38, 12.30, 17.22, 22.14 msec, FA = 20°. Fat saturation and double‐inversion black blood preparation were used.

The STEAM spectroscopy experiment used^28^: TE = 10 msec, TM = 7 msec, TR = 2 seconds for water‐suppressed spectra and TR = 4 seconds for water‐unsuppressed spectra and voxel volume was 8 cm^3^. The voxel of interest was placed in the lateral part of the right lobe of the liver, avoiding vessels, bile ducts, and the edge of the liver. Spectra were processed using AMARES in jMRUI^29^ with a specialized MatLab script^28^ and the fat fraction was determined as the ratio of the fat signal and the sum of the fat and water signals as in Eq. (1).

## Results

### Simulation

**Figure 3:**
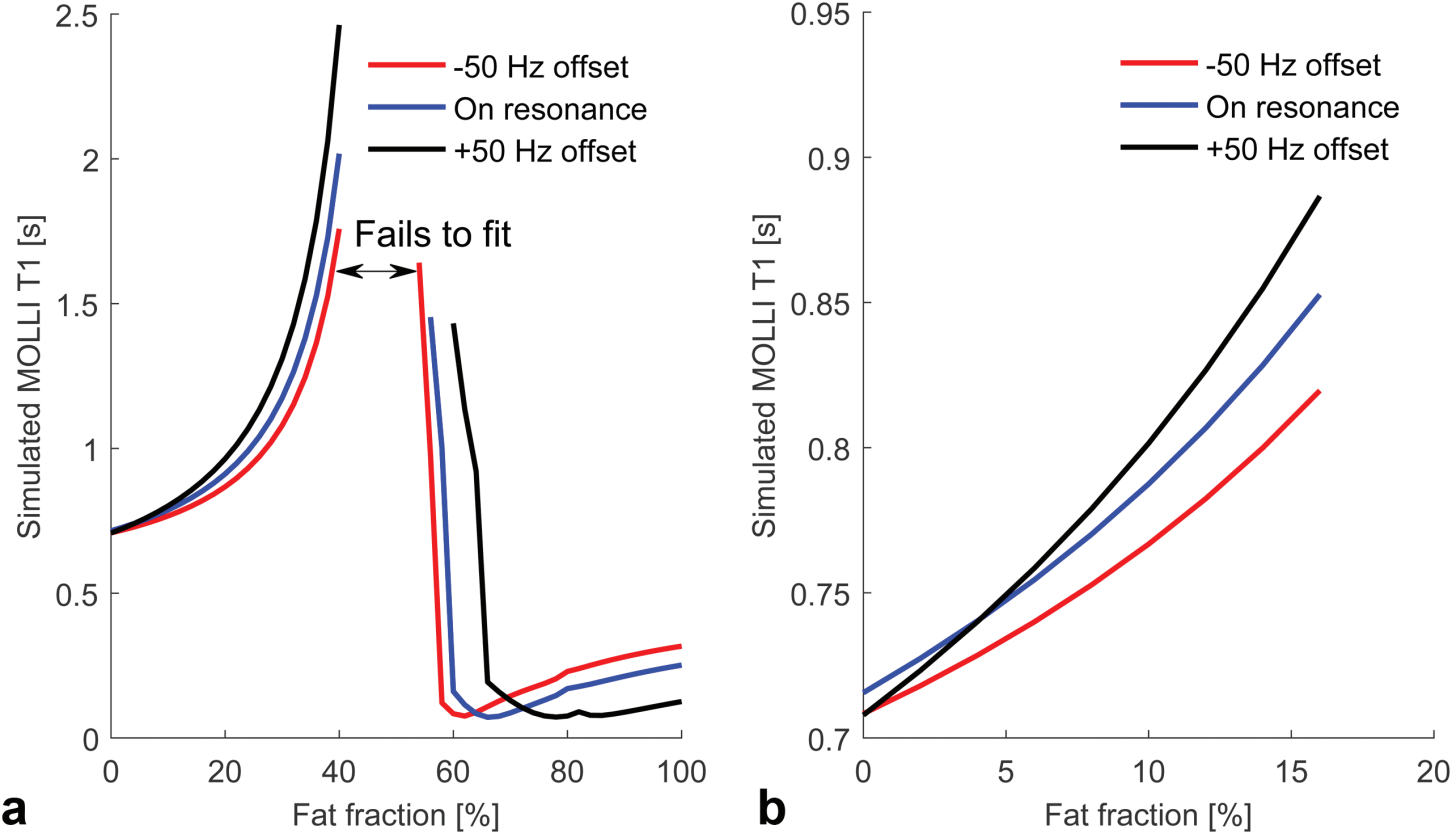
Variation of simulated MOLLI *T*_1_ values of the liver with fat fraction emphasizing behavior at off‐resonance: **(a)** global behavior of MOLLI *T*_1_ values over the full range of fat fractions; **(b)** MOLLI *T*_1_ behavior corresponding to the 0–16% range of fat fractions.

Figure 3 presents the relationship between the simulated MOLLI *T*_1_ and HLC. At low HLC (0–16% range) the measured MOLLI *T*_1_ increases with increasing fat, and can be approximated by:

(5) $$MOLLI\;T_{1}=715+5.47F+0.27F^{2}$$

where MOLLI *T*_1_ is expressed in milliseconds and *F* is the percent HLC. This trend ends at approximately *F* = 40%, where fitting becomes impossible owing to the water signal and fat signal canceling each other out. For *F* > 50% the MOLLI *T*_1_ values change rapidly, eventually reaching the *T*_1_ of the fat at 100% fat fraction. The increase of MOLLI *T*_1_ is explained by the simultaneous increase of both the B/A ratio and the apparent *T*_1_ (*T*_1_*) in Eq. (3).

**Figure 4:**
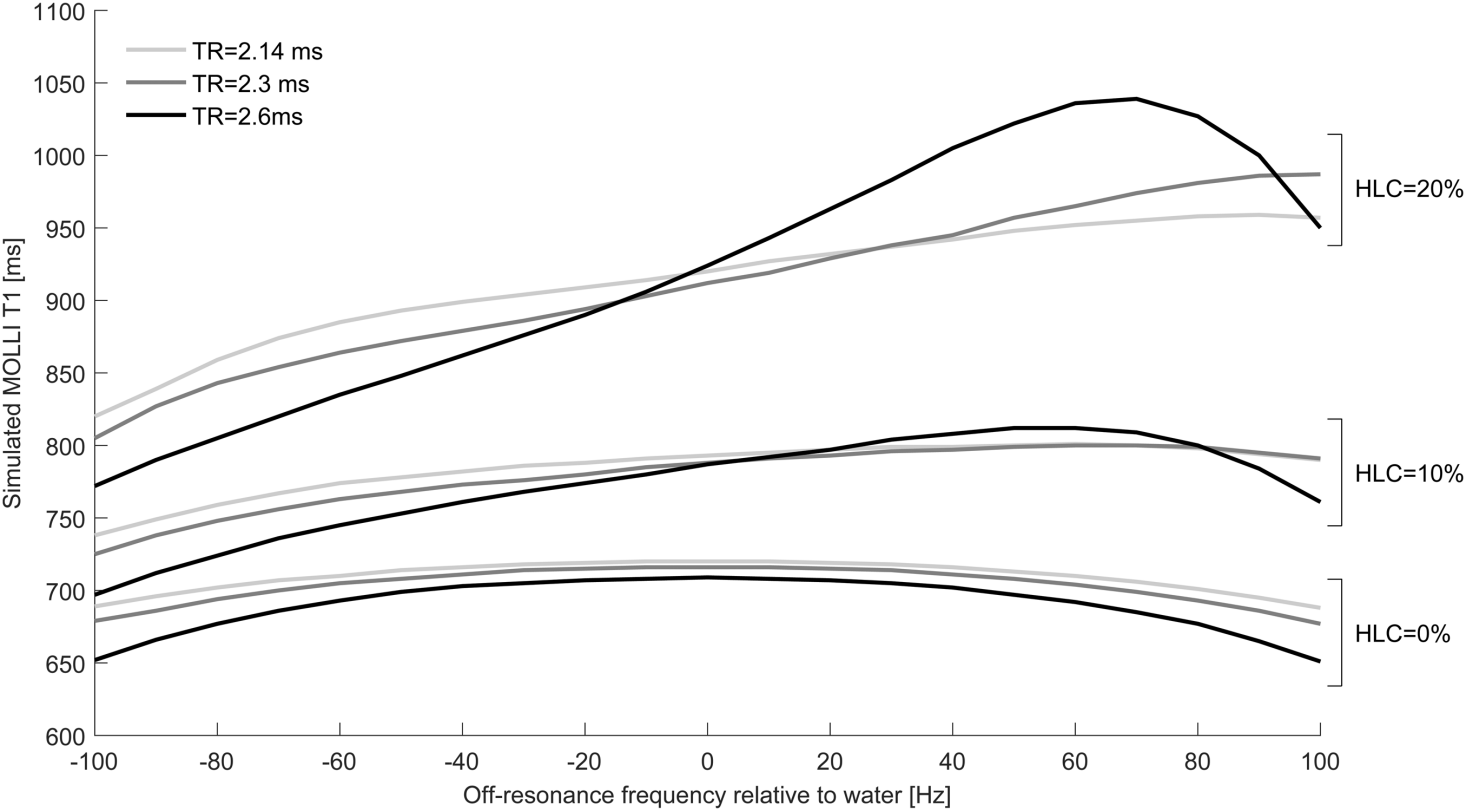
Variation of simulated MOLLI *T*_1_ with off‐resonance frequency and using repetition times described in the literature.^3^, ^13^

Figure 3 also presents the behavior of MOLLI *T*_1_ variation in the case of off‐resonance. Although the bSSFP readout leads to a symmetric MOLLI *T*_1_ variation around the central water frequency for 0% HLC, using a multiple‐peak spectral model of the lipid leads to asymmetric off‐resonance frequency dependence.

In simulations the dependence of MOLLI *T*_1_ on off‐resonance frequency when using repetition times from the literature^3^, ^13^ is shown in Fig. 4. Simulated MOLLI *T*_1_ values were found to be higher at lower repetition times for most fat fractions and off‐resonance frequencies. In addition, MOLLI *T*_1_–off‐resonance frequency dependence is both larger and increasingly asymmetric with respect to frequency in the presence of higher fat.

**Figure 5:**
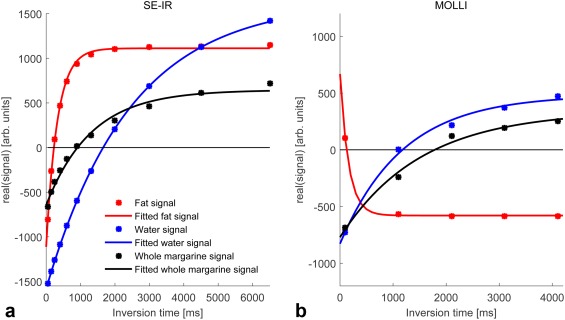
Fitted *T*_1_ recovery curves of whole margarine, fat component, and water component in the 30% fat margarine phantom: **(a)** shows the results of the spin echo inversion recovery experiment; **(b)** shows the results of the MOLLI experiment.

### Phantom Scans

*T*_1_ values determined from MOLLI and SE‐IR experiments in the margarine phantom are presented in Table 1.

**Table 1:** T_1_ Values Measured by the MOLLI Method and Computed From SE‐IR Images

	30% margarine	
Phantom type	MOLLI T_1_ [msec]	SE‐IR T_1_ [msec]
Whole margarine	3874 ± 177	1313 ± 14
Fat component	291 ± 23	325 ± 20
Water component	2266 ± 21	2448 ± 7

Figure 5 shows the signal intensities taken from regions of interest on SE‐IR images and the curves fitted to these points, along with the signal intensities sampled from MOLLI acquisitions and corresponding fitted curves.

**Figure 6:**
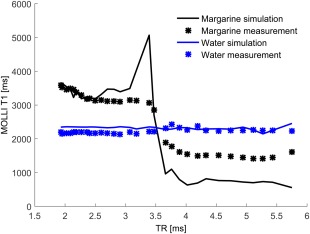
Variation of MOLLI *T*_1_ with the change of TR. Fat and water are out of phase at TR = 2.3 msec and in phase at TR = 4.6 msec.

The results of the simulation and phantom experiment exploring the dependence of MOLLI *T*_1_ on repetition times is shown in Fig. 6. Both simulated and measured MOLLI *T*_1_ values of the margarine phantom with 30% fat were higher than those of water over the 1.8 to 3.5 msec range and lower over the 3.5 to 5.75 msec range due to the relative position of the bSSFP profiles of the fat and water. However, the simulated and measured data did not agree perfectly. A possible explanation for the mismatch could be the missing information on the different *T*_1_ and *T*_2_ values of the individual fat peaks in the margarine phantom. A similar difference between simulation and phantom experiments can be seen in Thiesson et al's work.^11^

**Figure 7:**
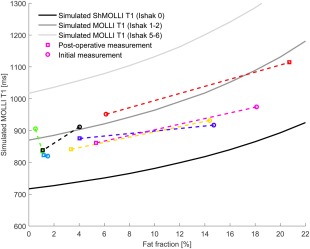
Continuous lines represent the variation of simulated MOLLI *T*_1_ with HLC, for three levels of liver fibrosis as defined by the Ishak score. These curves were obtained by running the simulation for *T*_1_ values corresponding to different disease states as described by Banerjee et al^3^ (see their fig. 2). Dashed line segments connect patient data points, showing the change in measured MOLLI *T*_1_ for each individual.

### Patient Studies

One patient was excluded from the comparison because the *T*_1_ fit to the MOLLI data failed at the first visit. The measured HLC for this patient was 39.7%, which is in the regime where the Bloch simulated data could also not be fitted to an inversion recovery model.

Six patients had normal *T*_2_* values larger than 19 msec and one had *T*_2_* = 15 msec, indicating elevated iron but all within the normal range for *T*_2_* at 3T.^30^

Pre‐ and postoperative MOLLI *T*_1_ measurements for seven patients are shown in Fig. 7, plotted as a function of their spectroscopically measured HLC. Simulated relationships between *T*_1_ and HLC are also included for three different levels of liver fibrosis, characterized by the Ishak score.^31^ MOLLI *T*_1_ values at 0% HLC for the three curves were taken from Banerjee et al, as shown in their fig. 2 of Ref. 3. The changes in MOLLI *T*_1_ that occur in these patients are consistent with the hypothesis that the water in the livers of these patients is elevated but stable between exams and that the fat concentration has changed. These patients are expected to have some level of inflammation or fibrosis, which has previously been shown to elevate MOLLI *T*_1_.^3^

## Discussion

In a physiological range of HLC from 0% to 45%^7^, ^8^, ^9^ we have shown that the MOLLI *T*_1_ at 3T increases with fat concentration in simulation and phantoms when using TR = 2.3 msec. This also applies when TR lies in the range 2.1 to 2.6 msec. The MOLLI *T*_1_ elevation is seen in vivo with the caveat that there may be other changes in the livers of the patients over 6 months after bariatric surgery that would be expected to modify the *T*_1_.^3^

Due to a chemical shift difference of 447 Hz between fat and water at 3T, the two tissue components are exactly out of phase with their passbands overlapping when using TR = 2.3 msec. Thus, combined signals from tissue containing fat and water are less dependent on off‐resonance frequency than reported previously using TR = 2.7 msec at 1.5T.^11^ Increasing or decreasing the TR from 2.3 msec at 3T reduces the overlap of the two bSSFP passbands, increasing the off‐resonance frequency dependence.

These simulations suggest that careful shimming, in addition to the use of a TR of 2.3 msec or less, is useful to maximize the consistency of MOLLI *T*_1_ measurements within a single subject and between subjects.

In contrast to 3T, where the optimal TR for reducing the frequency dependence of MOLLI *T*_1_ measurements is close to the minimum TR available with existing hardware, at 1.5T longer TR values would have to be used to produce a similar overlapping of the two bSSFP passbands due to the smaller chemical shift difference between fat and water.^32^

Patient MOLLI *T*_1_ evolution in a 6‐month interval after bariatric surgery follows our model describing the influence of fat on MOLLI *T*_1_ values. We expect that, while the amount of fat changes in these patients, other parameters known to affect the *T*_1_ (such as fibrosis and inflammation) remain fairly constant. In general terms, the change in measured MOLLI *T*_1_ is consistent with the behavior that is predicted by the simulation for changes in liver fat in five of the seven patients; in one patient the change in liver fat is very small and the MOLLI *T*_1_ change is small, and in one patient the liver fat change is small, but the MOLLI *T*_1_ change is ∼100 msec, not following the model. We believe that this supports the applicability of the model in vivo.

A limitation of this study is the small size of the patient cohort. A change in MOLLI *T*_1_ in two of the patients was not explained by change in fat fractions, suggesting the existence of other mechanisms responsible for having an influence on hepatic MOLLI *T*_1_ values.

The MOLLI *T*_1_ mapping method is used extensively in cardiac imaging and so it is important to briefly consider the effect of myocardial fat on MOLLI *T*_1_ maps at 3T. Since global lipid fractions in the myocardium are in the 0.2–2% interval,^28^, ^33^ they have a much smaller effect on measured MOLLI *T*_1_ values. An exception is in focal lipid accumulations, which can be as high as 35% in the case of lipomatous metaplasia,^12^ leading to replacement of scar tissue with lipid accumulations after myocardial infarction. Effects of these focal lipid concentrations are described by Kellman et al.^12^

In conclusion, this study has shown that the presence of fat influences MOLLI *T*_1_ measurements in the liver. This effect is in addition to the previously known effects of *T*_2_, magnetization transfer, off‐resonance frequency, and iron concentration. Simulation has shown that fat fractions up to 40% will have an additive effect on the measured MOLLI *T*_1_ value at 3T when using a bSSFP readout with TR = 2.3 msec. This behavior has been confirmed in the livers of patients undergoing weight‐reduction surgery.

The influence of fat should be considered in the assessment of hepatic diseases using MOLLI *T*_1_ measurements, as fat fraction values measured in the liver can be large enough to cause severe MOLLI *T*_1_ alterations.
